# Association between Phase Angle and Body Composition of Children and Adolescents Diagnosed with HIV Infection

**DOI:** 10.3390/children10081309

**Published:** 2023-07-29

**Authors:** Priscila Custódio Martins, Luiz Rodrigo Augustemak de Lima, Analiza Mónica Silva, Diego Augusto Santos Silva

**Affiliations:** 1 Research Center in Kinanthropometry and Human Performance, Department of Physical Education, Sports Center, Federal University of Santa Catarina, University Campus—Trindade-n. 476, Florianópolis 88040-900, Santa Catarina, Brazil; priscilaamartinsc@gmail.com; 2 Institute of Physical Education and Sport, Federal University of Alagoas, Campus A.C. Simões, Maceió 57072-900, Alagoas, Brazil; luiz.lima@iefe.ufal.br; 3 Exercise and Health Laboratory, CIPER, Faculdade Motricidade Humana, Universidade de Lisboa, 1499-002 Lisboa, Portugal; analiza@fmh.ulisboa.pt

**Keywords:** body composition, bone and bones, physical fitness, acquired immunodeficiency syndrome

## Abstract

The aim of the study was to investigate how phase angle (PhA) is associated with subtotal and lumbar spine bone mineral density [BMD], lean soft tissue mass [LSTM], total body fat mass, android and gynoid in children and adolescents with HIV according to sex. A cross-sectional study was conducted in Florianópolis, Brazil, involving 64 children and adolescents vertically transmitted with HIV. Resistance and reactance values were obtained using bioelectrical impedance analysis, and PhA was subsequently calculated. Dual emission X-ray absorptiometry was used to assess body composition. Antiretroviral medication, physical activity (accelerometers) and skeletal maturation (wrist-carpal radiography) were used in the adjusted model. In males, PhA was directly associated with subtotal BMD (βadj: 0.65; R²: 0.38, *p* < 0.01) and lumbar spine BMD (βadj: 0.53; R²: 0.22, *p* = 0.01), directly associated with LSTM (βadj: 0.76; R²: 0.46, *p* < 0.01), and inversely associated with gynoid fat (βadj: −0.47; R²: 0.2, *p* = 0.01), in adjusted models. In females, PhA was directly associated with subtotal BMD (βadj: 0.46; R²: 0.17, *p* < 0.01) and lumbar spine BMD (βadj: 0.48; R²: 0.19, *p* < 0.01). It is concluded that PhA was directly associated with subtotal and regional BMD, LSTM, and inversely with gynoid fat in boys with HIV. In girls, PA was directly associated only with subtotal and regional BMD.

## 1. Introduction

Children and adolescents diagnosed with HIV infection may have changes in body composition, mainly due to the deleterious effects of the virus and the continuous and prolonged use of drugs for antiretroviral therapy (ART) [1]. A study conducted with this population identified lower bone mineral density (BMD) and alterations in bone microarchitecture [2]. The literature has described that HIV infection itself can induce an increase in resorption and a decrease in bone formation. These factors result in a decrease in bone mass gains during growth. Furthermore, the pediatric population with HIV may have lipodystrophy syndrome (loss and/or accumulation of body fat) and loss of muscle mass, which negatively affects their health and quality of life [3,4]. In addition, research with children and adolescents diagnosed with HIV found that this population had delayed sexual maturation, which is mediated by physical growth variables [5]. Therefore, physical activity has been suggested as a non-pharmacological treatment to minimize the deleterious effects of the infection and improve their quality of life. However, a systematic review compiled from previous studies observed that Brazilian children and adolescents diagnosed with HIV often do not meet physical activity guidelines [6].

The phase angle (PhA) is obtained from the relation between resistance and reactance values (raw data from bioelectrical impedance analysis). The PhA is considered a marker of cell health and integrity and indicates the distribution of water between the intracellular and extracellular spaces [7]. PhA values above seven degrees suggest an adequate cellular integrity, while values below this threshold can indicate cellular damage [8]. In the context of patients living with HIV, PhA has been directly associated with infection prognosis [9] and mortality in adults [10]. Moreover, adults with HIV have shown lower PhA values compared to those without infection [11]. The reasons for the lower PhA values in this population are not fully understood, but studies have indicated that a lower PhA is directly associated with increased subcutaneous adipose tissue, and depletion of both muscle mass and body mass [9,12]; Additionally, the literature has shown that factors such as age [13], sex, body dimensions, biological maturation [14], physical activity level [15], and hydration status can directly impact PhA values in different age groups [16] and in individuals with different health conditions [9]. Therefore, these factors should be considered in the interpretation of the results found.

Previous works have evaluated the association between PhA and body composition, particularly in adults [17,18,19]. In the pediatric population without disease diagnoses, a study with adolescents identified a direct association between PhA and fat-free mass in both sexes, and a direct association with relative body fat only in females, adjusted for peak height velocity [20]. In children and adolescents aged 9 to 11 years, a study found that in females, PhA was inversely associated with total body fat and directly associated with fat-free mass. In males, PhA was not associated with body composition [20]. In HIV+ children and adolescents, we found only one study, our own [21]. In this study, we observed a direct association between PhA and lean soft tissue mass (LSTM) in children and adolescents of both sexes, while PhA was not associated with relative or absolute body fat [21]. However, in this study, we did not perform sex-stratified analyses, which may have directly impacted the results. Regarding the possible relationship between PhA and BMD, no studies were found in the literature with the pediatric population. However, in elderly people without a diagnosis of the disease, an association was observed between PhA and BMD (total and regional) [17].

A scoping literature review found that the PhA was directly associated with lean mass and muscle mass in different age groups [22]. However, the review strongly emphasized the presence of the following gaps regarding the relationship between PhA and body composition in the pediatric population: (1) There was no consensus in the literature about the direction of PhA associations with fat mass and (2) Only one study evaluated the association between PhA and BMD in adults [22].

Despite the literature presenting studies that have assessed the relationship between PhA and body composition, the physiological mechanisms have not been fully elucidated, especially in the population diagnosed with HIV. Furthermore, the evaluation of body composition is complex because it involves various components and levels of analysis and each component influences health through specific biological mechanisms [23]. Additionally, certain mechanisms directly affect body composition, such as HIV infection. Lastly, considering biological maturation and physical activity level is important in the context of HIV, due to the impacts of the infection mentioned earlier.

Therefore, the following research problem was formulated: what is the association (magnitude and direction) between PhA and body composition in the pediatric population with HIV? To answer the research problem, the aim of the study was to investigate whether PhA is associated with body composition components [BMD (subtotal and lumbar spine), LSTM, total and regional body fat mass (android and gynoid)], even after adjusting for bone age, physical activity, and ART in HIV+ children and adolescents according to sex.

## 2. Method

The method used in this article followed the procedures recommended by strengthening the reporting of observational studies in epidemiology (STROBE-checklist).

### 2.1. Study Design

The present study is a cross-sectional analysis conducted in 2016 in the city of Florianópolis, Santa Catarina, Brazil. The Research Ethics Committee of the Federal University of Santa Catarina approved the study protocol under the opinion 49691815.0.0000.0121 on 15 February 2016. All parents/guardians of the children and adolescents signed the informed consent form authorizing their participation in the research. The participating children and adolescents also signed an assent form. 

### 2.2. Population and Sample

**Figure 1:**
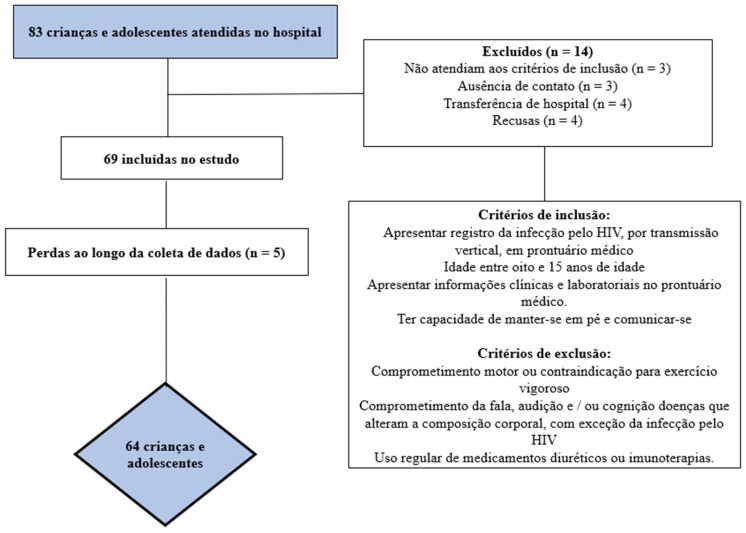
Flowchart of study participants.

The sample recruitment process is described in Figure 1. After identifying the total number of eligible children and adolescents, the sample power was calculated. To calculate the a posteriori sample, the G*Power^®^ software version 3.1.9.2 (Universität Düsseldorf, Düsseldorf, Germany) was used with the parameters β = 0.80 and α = 0.50 [24]. With a sample size of at least 55 participants, it was possible to find effect associations of at least 0.50 (average effect) [25].

### 2.3. Phase Angle

The analysis of PhA was performed using bioelectric impedance (BIA) InBody^®^ 720 model, a multi-frequency octopolar device (Biospace, Los Angeles, CA, USA), which provided impedance (Z) and reactance (Xc) data. From these variables at a frequency of 50 kHz, the resistance (R) value was calculated, considering body size [26]. The formula used to calculate PhA was arctangent (Xc/R) × 180°/π [9].

During the evaluation, the participants remained standing on a platform. After the equipment measured body mass, the participants were instructed to hold the handles with their arms away from their torso. The entire procedure took approximately two minutes. All participants were instructed to follow the pre-test recommendations, including fasting for at least four hours, wearing appropriate clothing, being barefoot, refraining from wearing earrings and/or rings, abstaining from intense physical activity on the previous day, avoiding beverages with high caffeine content in the previous 12 h and not be on their menstrual period. If the child and/or adolescent was menstruating, the evaluation was rescheduled. Test–retest reproducibility for resistance and reactance was examined in a similar independent sample in terms of age and sex (*n* = 7). In this way, the intraclass correlation coefficient was calculated for R = 0.82 [95% confidence interval (95% CI): 0.81–0.97], and Xc = 0.91 (95% CI: 0.90–0.98).

### 2.4. Body Composition

Dual-energy X-ray absorptiometry (DXA) (GE^®^ Lunar Prodigy Advance, GE Medical Systems, Madison, WI, USA) was used to assess body composition using ENCORE 13.60.033 software. The biometric scan using a specific whole-body sensor took approximately six minutes while the participant lay in the supine position. The DXA assessment was conducted by a researcher trained in equipment operation using standardized procedures [27]. The following components were utilized in this study based on the DXA results: subtotal BMD (excluding the head) and lumbar spine (L1–L4) BMD, total body fat percentage, android fat percentage, and gynoid fat percentage. Additionally, by measuring bone mass and distinguishing bones from adipose tissue and fat-free mass, LSTM was estimated by subtracting these components [28]. 

For the calculation of android fat percentage, DXA identifies regions of interest (ROIs) with the lower edge at the pelvic cut, the upper edge above the pelvic cut at 20% of the distance between the pelvic and neck cuts, and the lateral edges at the arm cuts [24]. For the calculation of gynoid fat percentage, DXA identifies ROIs with the upper edge below the pelvic cut line at 1.5 times the height of the android ROI, and the height of the gynoid ROI is equal to two times the height of the android ROI, with the lateral edges at the outer leg cuts [27].

For DXA assessment, it is necessary to measure body mass and standing height. Body mass information was obtained using BIA (InBody^®^ 720, a multi-frequency octopolar device, Biospace, Los Angeles, CA, USA). Height was measured following the protocol of the International Society for the Advancement of Kinanthropometry (ISAK) by a level 1 researcher, using an AlturaExata^®^ stadiometer (Belo Horizonte, Brazil), with a resolution of 1 mm. Test–retest reproducibility for total body fat was examined in a similar independent sample in terms of age and sex (11.6 ± 5.8 kg and 11.5 ± 5.9 kg; intraclass correlation coefficient = 1.00; 95% CI: 0.99–1.00; *n* = 10) [29].

### 2.5. Covariates

Information regarding ART, categorized as “no ART”, “ART without protease inhibitors (PIs)”, and “ART with PIs”, as well as sex, was obtained from medical records. Skeletal maturation was assessed using left hand wrist-carpal radiographs conducted at the Radiology Department of HIJG. The procedures followed standardized protocols described in the literature [30]. Children and adolescents were classified into three categories based on the difference between chronological age and bone age. If the bone age of a participant was advanced by more than one year compared to chronological age, it was classified as “early”. If the bone age was delayed by more than one year compared to chronological age, it was classified as “late”. If the difference between chronological age and bone age was within the range of −1 to +1, it was classified as “normal” [31].

Physical activity of moderate to vigorous intensity (MVPA) was assessed using an Actigraph^®^ accelerometer (Manufacturing Technology Inc., Fort Walton Beach, FL, USA), model GT3X-Plus, worn continuously for seven to 14 days, including weekends [32]. Participants were required to wear the device from the morning until the end of the day, removing it only during water-based activities and sleep [33]. Data analysis included records from a minimum of four days (three weekdays and one weekend day) with a minimum wear time of 10 h, after removing periods of at least 60 consecutive zeros indicating non-wear time. MVPA minutes were determined using cut-off points described by Evenson et al. [34] and adjusted proportionally to the average waking time of adolescents (14 h). 

### 2.6. Statistical Analysis

First, descriptive analysis was performed using median and interquartile range. Kurtosis and skewness were assessed to verify data normality (within the range of −2 to +2), along with histogram analysis to identify normal distribution of the data. Independent samples *t*-test was employed, and Cohen’s d effect size was calculated to classify effect sizes as small (d = 0.2), medium (d = 0.5), or large (d ≥ 0.8) [35]. A chi-square test was used with Cramer’s V effect size, ranging from 0 to 1, where a value of 0 indicates no association between variables, while values closer to 1 indicate a strong association between variables [36].

Pearson’s linear correlation was used to identify correlations between PhA and body composition parameters. Simple linear regression and multiple linear regression were employed, and two models were built for all parameters of body composition. Model 1 included only the PhA variable and each component of body composition. Model 2 included PhA, body composition component, and additional covariates such as skeletal maturation, ART, and MVPA. Regression coefficients (β), both simple and standardized, 95% CI, and the coefficient of determination (R²) were estimated for each analyzed model. Cohen’s d effect size was also calculated [35]. Model quality parameters, such as multicollinearity analysis, model residuals, Bayesian Information Criteria (BIC), and Akaike’s Information Criteria (AIC) were assessed and found to have values considered appropriate [34]. All analyses were performed using STATA software (Statistical Software for Professionals, College Station, TX, USA), version 14.0, with a significance level of *p* ≤ 0.05.

## 3. Results

The study had a total of 64 participants, with 35 being female. Males had a higher PhA value (mean: 5.18 ± 0.74 degrees) compared to females (mean: 4.84 ± 0.51 degrees) (*p* < 0.036). On the other hand, females had higher values of total body fat (mean: 10.44 ± 5.65 kg), android fat (mean: 26.33 ± 10.87%), and gynoid fat (mean: 37.74 ± 7.03%) compared to males (total body fat: 6.26 ± 3.28 kg); android fat: (mean: 17.84 ± 9.00%; gynoid fat: 26.01 ± 9.43%), with *p* ≤ 0.05. Regarding skeletal maturation, three participants (two boys and one girl) did not present data for this variable. Thus, of the 32 girls evaluated, it was observed that 10 (83.33%) presented “early” maturation and five (33.33%) presented “late” maturation. In comparison, two boys had “early” maturation (16.67%) and 10 (66.67%) had “late” skeletal maturation (*p* = 0.03) (Table 1).

**Figure 2:**
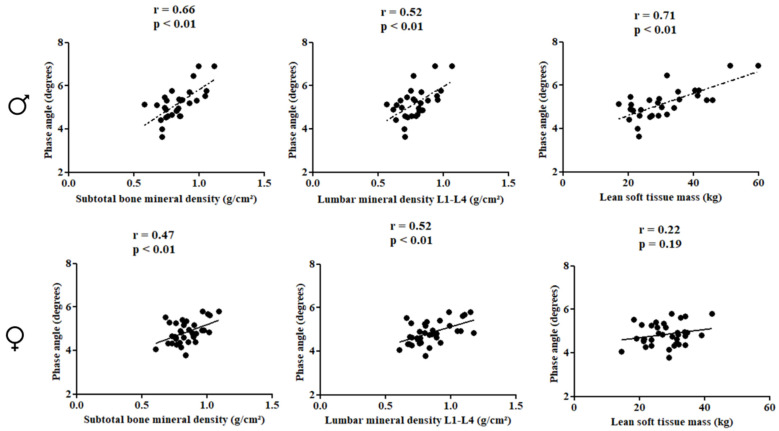
Correlation between the study variables in the pediatric population with HIV according to sex.

**Figure 3:**
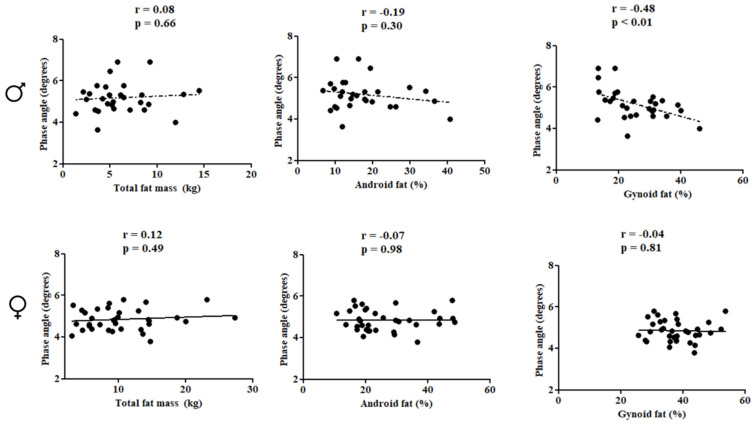
Correlation between the study variables in the pediatric population with HIV according to sex.

In females, PhA was directly correlated with subtotal BMD (r = 0.66; *p* < 0.01), lumbar spine BMD (r = 0.52; *p* < 0.01), and LSTM (r = 0.71; *p* < 0.01). In males, PhA was inversely associated with gynoid fat (r = −0.48; *p* < 0.01) (Figure 2 and Figure 3).

Multiple linear regression analyses showed that PhA was positively associated with subtotal BMD (βstd: 0.65; R²: 0.38, *p* < 0.01) and lumbar spine BMD (βstd: 0.53; R²: 0.22, *p* = 0.01), and positively associated with LSTM (βstd: 0.77; R²: 0.55, *p* < 0.01), while being inversely associated with gynoid fat in males (βstd: −0.41; R²: 0.30, *p*: 0.04), even after adjusting for covariates. For females, PhA was positively associated with subtotal BMD (βstd: 0.46; R²: 0.17, *p* < 0.01) and lumbar spine BMD (βstd: 0.48; R²: 0.19, *p* < 0.01) (Table 2).

## 4. Discussion

In the present study, PhA was directly associated with subtotal and lumbar spine BMD in a pediatric population with HIV in both sexes, even after adjusting for skeletal maturation, ART, and MVPA. Additionally, PhA was directly associated with LSTM and inversely associated with android fat in males.

Similar results to those of the present study have been observed in previous research investigating the association between PhA and total and regional BMD (lumbar spine, hip, femoral neck, neck, and forearm) However, these surveys were conducted with adults or older adults individuals, which makes it difficult to compare results. Although, no previous studies were located that investigated the association between PhA and BMD in children and adolescents [22]. In patients with chronic kidney disease, PhA was directly associated with femoral neck BMD, even after considering the effects of age, alkaline phosphatase, intact parathyroid hormone, and LSTM [37]. In older adults, PhA was directly associated with total BMD, femur BMD, and neck BMD, even after adjustment for covariates that included sociodemographic variables (e.g., age) and personal history of fractures [17]. In postmenopausal women, PhA was directly associated with total BMD and hip BMD only in the normal BMI group, while no association was found between PhA and total or hip BMD in women who are overweight or obese [38].

The mechanisms underlying the aforementioned relation have not been fully elucidated, but it is suggested that: (1) LSTM may provide increased mechanical loading on the bone because it leads to increased muscle strength (or vice versa) and, consequently, increased tensile load on muscles exerted on bones. This mechanical stress stimulates bone formation (osteoblasts and osteocytes) [39]; (2) Bones are made up of cellular components, a mineral component, and an organic matrix. Thus, there seems to be an association between bone composition and electrical conductivity [40,41]. Therefore, bones can generate electrical conductivity which affects the body’s resistance values and since PhA is calculated from resistance and reactance values, it can also be impacted. Thus, the current results demonstrate that PhA was directly associated with subtotal and lumbar spine BMD which are in line with these proposed mechanisms.

Regarding the direct association between PhA and LSTM observed in the present study for males, similar results have been observed in adolescents and adults without diagnosed diseases [20,42]. However, a large proportion of studies have assessed fat-free mass rather than LSTM [20,42]. LSTM, as obtained by DXA, represents the sum of body water, protein, non-bone minerals (sodium, potassium, chloride, hydrogen phosphate, and bicarbonate), and glycogen [41]. A study conducted with adolescents without diagnosed diseases of both sexes identified a direct association between PhA and fat-free mass, even after adjusting for peak height velocity [20]. In adults without diagnosed diseases of both sexes, direct associations between PhA and fat-free mass, assessed by hydrostatic weighing, were also observed, even after adjusting for skin color, age, body mass, height, and BMI [42]. Although both studies [20,42] controlled for relevant confounding variables in their analyses, the role of physical activity level was not explored in either study. Furthermore, Gonzalez et al. [42] included other variables directly related to body composition, such as body mass and BMI, which may lead to issues of multicollinearity in the analyses.

In females we found that PhA was not directly associated with LSTM. The literature has shown that PhA differs between sexes. The justifications discussed in the literature are mainly based on the presence of greater muscle mass in males compared to females [23]. However, it is important to extrapolate to other factors, such as changes in body water, especially during the maturation period, and body size (height) [13]. Therefore, it is believed that it is important to consider sex-stratified analyses, which can help understand the differences in physical growth, body composition, and lifestyle between sexes reported in the literature [23]. In addition to sex, the literature has reported that other factors directly impact PhA, such as age, physical activity level [15], biological maturation [13], and specific aspects of each investigated population, such as disease stage and medication use [7,9]. Age shows a particular behavior with PhA, as it increases until adulthood but tends to decrease with advancing age, mainly due to changes in physical growth and body composition [9]. Regarding physical activity, a systematic review with meta-analysis compiled different studies that observed higher PhA values in physically active individuals and athletes, demonstrating the impact of physical activity on PhA [15].

In this study, we also investigated the relationship between PhA and gynoid fat and android fat. We chose to conduct this investigation for the following reasons: Children and adolescents HIV+ may have alterations in the pattern of body fat distribution, known as lipodystrophy [43] and Body fat located in the trunk region is recognized in the literature for its high inflammatory potential, directly contributing to deleterious health effects [44]. PhA was inversely associated with gynoid fat in males. Gynoid fat is typically located in the hip region, and females generally have higher gynoid fat compared to males [45]. This result was also observed in the present study, where higher values of gynoid fat were found in females. Previous research has investigated the association between PhA and total body fat, but only one study examined the relationship with visceral fat in adults without diagnosed diseases and found an inverse association [46]. Investigating body fat in specific body regions is important in the context of HIV, particularly due to potential alterations in fat distribution patterns, with abdominal fat predominance and pronounced fat loss in the extremities (arms and legs) [22]. The inverse association between PhA and body fat seems to be associated with the electrical conductivity of body tissues, which are different depending on the constitution of the tissues. Body fat, for example, has low electrical conductivity, but high resistance to current flow, resulting in high resistance values. Additionally, body fat triggers inflammatory processes that can damage cell membranes [47,48] thereby decreasing reactance values.

The use of DXA can be seen as a strong point of this study, the research team underwent rigorous training and all the standardized procedures were followed to ensure data reliability. Additionally, the analysis of MVPA using accelerometers allowed for the inclusion of this variable as a potential confounder in the association between PhA and BMD. However, this study also has limitations. The heterogeneity of the sample resulting from variations in age range, clinical condition, and ART regimen constitutes a limitation. Due to the limited sample size, it was not feasible to conduct stratified analyses based on these variables. However, in an effort to mitigate the influence of age and ART regimen, we included bone age and ART use as covariates in the analyses. It is worth noting that according to a recent literature review [22] no previous studies have investigated the association between PhA and BMD in pediatric populations. Consequently, comparisons with the existing literature were drawn from studies conducted with adult populations, potentially leading to erroneous interpretations. 

Based on the findings, it can be concluded that PhA was directly associated with subtotal and regional BMD (lumbar spine) in children and adolescents with HIV diagnosis. Moreover, PhA was directly associated with LSTM and inversely associated with gynoid fat in males, even after adjusting for skeletal maturation, ART, and MVPA. Considering the ease of use of BIA, PhA can be employed in the planning, screening, and monitoring of the health of children and adolescents with HIV infection as an additional measure. However, caution should be exercised when interpreting the results, and further studies are needed to clarify the sexual dimorphism in the association between PhA and body composition in children and adolescents with HIV diagnosis.

From a practical perspective, the phase angle is an easily obtainable measure, and there are even portable BIA models with a relatively low financial cost. Thus, this study can contribute to the understanding of the use of the phase angle as an additional tool to obtain information on bone health and the distribution of total and regional fat mass in the pediatric population with HIV.

## Figures and Tables

**Table 1:** Characteristics of pediatric population with HIV.

	Male (*n* = 29)		Female (*n* = 35)			
	**Mean (±SD)**	**Min; Max**	**Mean (±SD)**	**Min; Max**	***p*-Value**	**Cohen’D**
Age (years)	12.24 (2.19)	08; 15.00	12.22 (2.09)	08; 15.00	0.97	0.01
Bone age (years)	11.74 (2.66)	06. 15.00	12.40 (2.73)	06. 17.00	0.34	0.24
Body mass (kg)	39.45 (12.24)	21.09; 72.40	40.38 (10.94	18.70; 65.10	0.758	0.08
Height (cm)	147.72 (13.78)	116.80; 173.10	147.00 (12.63)	114.20; 167.00	0.83	0.05
Body mass index (kg.m^−2^)	17.61 (2.67)	12.52; 24.16	18.31 (2.73)	14.33; 24.80	0.31	0.25
Resistance (Ω/m)	592.5.65 (119.01)	352.26; 815.61	640.22 (75.92)	468.62; 799.78	0.06	0.50
Reactance (Ω/m)	53. 36 (8.35)	41.23; 69.74	53.65 (7.95)	38.35; 69.63	0.87	0.03
Phase angle (degrees)	5.18 (0.74)	3.64; 6.91	4.84 (0.51)	3.79; 5.81	**0.03**	0.53
Lean soft tissue mass (kg)	31.00 (10.21)	17.00; 59.86	27.94 (6.13)	14.46; 42.30	0.14	0.36
Total fat mass (kg)	6.26 (3.28)	1.3 (14.41)	10.44 (5.65)	3.02 (27.27)	**<0.01**	0.88
Android fat (%)	17.84 (9.00)	6.70; 40.60	26.33 (10.87)	10.50; 48.60	**0.01**	0.84
Gynoid fat (%)	26.01 (9.43)	13.20; 46.10	37.74 (7.03)	25.60; 53.50	**<0.01**	1.42
Subtotal bone mineral density (g/cm²)	0.83 (0.12)	0.58; 1.11	0.84 (0.10)	0.60; 1.09	0.84	0.06
Lumbar mineral density L1–L4 (g/cm²)	0.78 (0.11)	0.56; 1.06	0.84 (0.15)	0.60; 1.17	0.06	0.47
Subtotal bone mineral content (g)	1150.81 (474.05)	394.40; 2462.70	1202 (434.03)	338.30; 2073.6	0.64	0.11
Lumbar mineral content L1–L4 (g)	119.17 (52.79)	46.90; 276.40	136.20 (59.72)	38.40 267.70	0.23	0.30
Viral load (log)	2241.72 (1042.78)	1602.00; 5040.00	2118. 34 (932.83)	1602.00; 4971.00	0.61	0.12
CD4 T lymphocytes (cells.mm^−3^)	861.50 (364.55)	196.00; 1811.00	854.31 (375.71)	135.00; 1731.00	0.93	0.09
CD8 T lymphocytes (cells.mm^−3^)	1204.25 (489.30)	402.00; 2698.00	1125.25 (580.90)	495.00; 3583.00	0.57	0.14
Moderate-vigorous physical activity (min.day)	48.87 (26.46)	12.50 141.80	47.24 (27.47)	10.50; 128.50	0.83	0.06
	***n* (%)**			***n* (%)**	***p*-value**	**V Cramér**
ART					0.18	0.16
Yes	26 (49.06)			27 (50.94)		
No	03 (27.27)			08 (72.73)		
Skeletal maturation *						
Early	02 (16.67)			10 (83.33)	**0.03**	0.32
Normal	16 (54.29)			19 (45.71)		
Late	10 (66.67)			05 (33.33)		

kg: kilograms; m: meters. f: strength; mL: milliliters; min: minutes; %: percentage: Ω: ohms; g: grams; cm: centimeters; mm: milliliters ART: antiretroviral therapy. Data in bold signify statistical significance. Data in bold signify statistical significance. Three children (boys) did not present skeletal maturation data. * Three children (two males and one female) did not show skeletal maturation data. Bold: *p*-value ≤ 0.05.

**Table 2:** Association of phase angle with body composition according to sex.

	Boys (*n* = 29)					Girls (*n* = 35)				
	β (CI95%)	β Pad	R²	*p*-Value	f²	β (CI95%)	β Pad	R²	*p*-Value	f²
Subtotal bone mineral density										
Simple model	3.93 (2.18; 5.67)	0.66	0.42	**<0.01**	0.72	2.19 (0.73; 3.65)	0.21	0.20	**0.02**	0.25
Adjusted model	4.04 (1.65; 6.16)	0.65	0.38	**<0.01**	0.61	2.23 0.63; 3.81)	0.46	0.17	**<0.01**	0.20
Lumbar mineral density L1–L4										
Simple model	3.59 (1.54; 5.64)	0.57	0.29	**<0.01**	0.40	1.76 (0.74; 2.78)	0.62	0.25	**<0.01**	0.33
Adjusted model	3.38 (0.74; 6.02)	0.53	0.22	**0.01**	0.28	1.71 (0.55; 2.86)	0.48	0.19	**<0.01**	0.23
Lean soft tissue mass										
Simple model	0.05 (0.03; 0.07)	0.71	0.49	**<0.01**	0.96	0.01 (−0.01; 0.04)	0.22	0.05	0.19	0.05
Adjusted model	0.05 (0.02; 0.84)	0.76	0.46	**<0.01**	0.85	0.01 (−0.01; 0.04)	−0.12	0.20	0.26	0.25
Total fat mass										
Simple model	0.02 (−0.07; 0.11)	0.08	0.01	0.67	0.01	0.01 (−0.02; 0.04)	0.12	0.01	0.49	0.01
Adjusted model	−0.02 (−0.10; 0.11)	0.01	0.01	0.96	0.01	0.01 (−0.02; 0.01)	0.09	0.01	0.61	0.01
Android fat										
Simple model	−0.01 (−0.04; 0.01)	−0.19	0.04	0.30	0.04	−0.01 (−0.04; 0.02)	−0.01	0.01	0.96	0.01
Adjusted model	−0.01 (−0.04; 0.02)	−0.26	0.01	0.51	0.01	0.01 (−0.01; 0.02)	−0.04	0.01	0.65	0.01
Gynoid fat										
Simple model	−0.04 (−0.06; −0.01)	−0.48	0.23	**<0.01**	0.30	−0.02 (−0.02; 0.02)	−0.04	0.01	0.81	0.01
Adjusted model	−0.03 (−0.07; −0.01)	−0.47	0.21	**0.01**	0.26	−0.01 (−0.02; 0.01)	−0.03	0.01	0.73	0.01

Adjusted model: skeletal maturation, physical activity and type of antiretroviral therapy. Bold: *p*-value ≤ 0.05.

## Data Availability

All data can be requested from the authors if necessary.
